# An Improved Two-Step Strategy for Accurate Feature Extraction in Weak-Texture Environments

**DOI:** 10.3390/s25206309

**Published:** 2025-10-12

**Authors:** Qingjia Lv, Yang Liu, Peng Wang, Xu Zhang, Caihong Wang, Tengsen Wang, Huihui Wang

**Affiliations:** School of Mechanical Engineering & Automation, Dalian Polytechnic University, Dalian 116034, China; lvqingjia456@163.com (Q.L.); wp1176407051@163.com (P.W.); zhangxu_dlut@163.com (X.Z.); 17866607600@163.com (C.W.); jackwang8555@foxmail.com (T.W.)

**Keywords:** weak-texture, binocular vision, 3D reconstruction, environmental perception

## Abstract

To address the challenge of feature extraction and reconstruction in weak-texture environments, and to provide data support for environmental perception in mobile robots operating in such environments, a Feature Extraction and Reconstruction in Weak-Texture Environments solution is proposed. The solution enhances environmental features through laser-assisted marking and employs a two-step feature extraction strategy in conjunction with binocular vision. First, an improved SURF algorithm for feature point fast localization method (FLM) based on multi-constraints is proposed to quickly locate the initial positions of feature points. Then, the robust correction method (RCM) for feature points based on light strip grayscale consistency is proposed to calibrate and obtain the precise positions of the feature points. Finally, a sparse 3D (three-dimensional) point cloud is generated through feature matching and reconstruction. At a working distance of 1 m, the spatial modeling achieves an accuracy of ±0.5 mm, a relative error of 2‰, and an effective extraction rate exceeding 97%. While ensuring both efficiency and accuracy, the solution demonstrates strong robustness against interference. It effectively supports robots in performing tasks such as precise positioning, object grasping, and posture adjustment in dynamic, weak-texture environments.

## 1. Introduction

**Figure 1:**
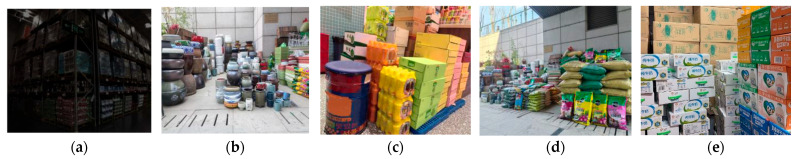
Weak-texture scenes in industrial environments. (**a**) Low brightness; (**b**) Uneven distribution of features; (**c**) Lack of texture; (**d**) Various shapes; (**e**) High texture similarity.

In the intelligent transformation of industrial scenarios, particularly in packaging and logistics, vision-driven mobile robots often operate in weak-texture environments. A weak-texture environment(e.g., Figure 1a–e) is characterized by a lack of prominent high-contrast texture features, typically exhibiting sparse textures, uniform colors (such as white walls, solid-colored objects, and dimly lit scenes), and difficulty in recognizing local features (such as edges and corners). In such environments, robots struggle with feature extraction, leading to inaccurate depth estimation, which in turn affects 3D localization and pose recognition, reducing operational stability.

For example, in food and beverage warehouses with low light and monotonous backgrounds (e.g., walls, columns, and supports), robots face difficulties in handling featureless or solid-colored beverage boxes, often failing to acquire complete 3D scene information. Traditional perception methods struggle to extract features effectively, resulting in environmental perception errors. These errors not only impact path planning and operational accuracy but can also lead to collisions, product damage, or misplacement, increasing operational risks. The widespread presence of weak textures further exacerbates the challenges to robot adaptability and stability in complex dynamic environments.

Existing solutions are generally categorized into two types: passive vision and active vision. Passive vision technology [1,2,3,4,5,6,7,8,9,10,11,12,13] captures environmental images using sensors such as cameras and extracts environmental information through image processing and analysis. Although it offers simplicity, compactness, and low cost, it remains challenging to extract effective features in environments characterized by low texture, poor lighting, or motion blur. Active vision technology [14,15,16,17,18,19,20,21,22,23,24,25,26,27,28] acquires environmental information by emitting signals and analyzing their reflections. Common devices include LiDAR and ultrasonic sensors. While this method demonstrates strong adaptability to lighting conditions and provides high-precision environmental data, it also suffers from drawbacks such as high equipment and maintenance costs, high computational load, and limited real-time capability.

**Figure 2:**
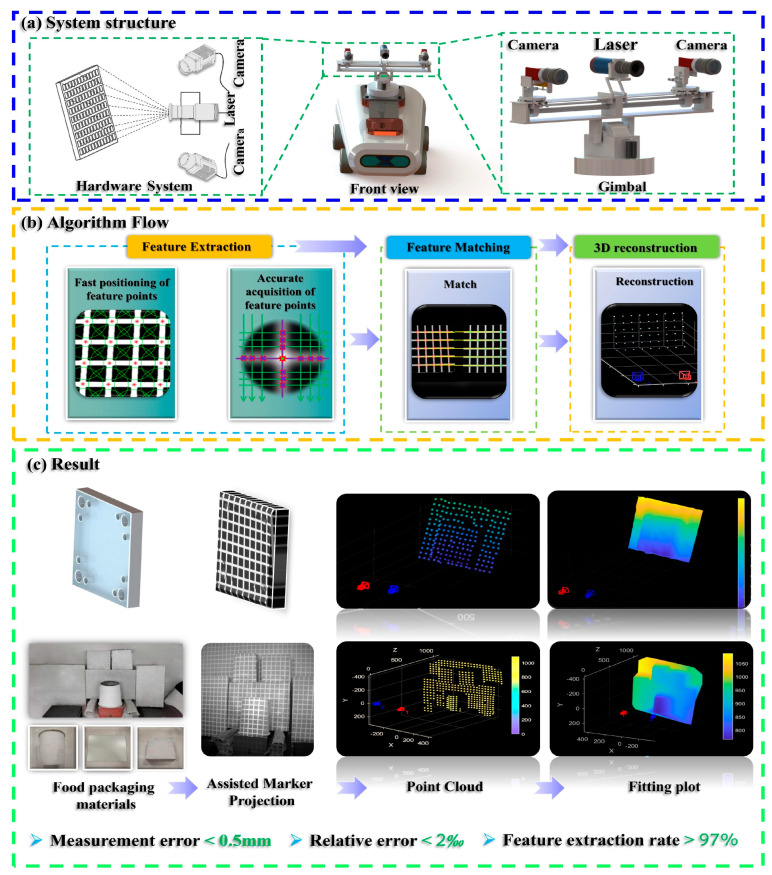
The main flow of the research: (**a**) System structure; (**b**) Algorithm Flow; (**c**) Result.

In response to the challenges mentioned above, this study proposes an environmental perception solution based on sparse point cloud reconstruction, integrating binocular vision with laser-assisted marking. The method is designed to enhance 3D perception in complex industrial scenarios characterized by weak textures and Gaussian blur, aiming to achieve fast, accurate, and robust environmental understanding for robots operating under such conditions. This solution features low cost, minimal computational resource consumption, and a compact structure, while also enabling fast and accurate positioning and operation for compact, low-cost robots in texture-deficient environments. The specific components of the proposed method are outlined as follows:

- To address the challenge of acquiring feature information in weak-texture scenes, a vision–laser fusion-based solution has been proposed, as shown in Figure 2. This method involves designing projected auxiliary markers and constructing an environmental model based on their discrete distribution. Additionally, a two-stage strategy is employed, first rapidly locating and then precisely extracting feature points, thereby balancing efficiency and accuracy.
- To overcome the challenge of rapid feature point localization in complex environments, a multi-constrained enhanced SURF algorithm has been developed. This method integrates grayscale intensity and morphological features to improve both the efficiency and reliability of feature point detection.
- To address the challenge of precise feature point extraction, a method integrating the Steger algorithm with a weighted fitting model is proposed. Building on rapid localization and incorporating the principles of laser imaging and optical characteristics, this method enables high-precision extraction of feature points.

The comparative analysis with commercial counterparts in Table 1 demonstrates that the proposed 3D perception method for weak-texture environments innovatively adopts a “laser-assisted marking—improved SURF feature extraction—Steger subpixel correction” technical chain, achieving a measurement accuracy of ±0.5 mm at a 1-m working distance. Compared with existing systems, this approach significantly reduces hardware costs while maintaining a compact structural design.

The laser projector used in the present study differs from commercial counterparts in that its primary function is simply to project simple patterns onto the environment. As a result, the performance requirements for the laser projector are relatively low, leading to a reduced cost and a very compact structure.

By effectively addressing perception challenges caused by weakly textured surfaces, low illumination, and dynamic interference in robotic operation scenarios, this solution provides mobile robots with a 3D perception system that combines high precision, low cost, and easy deployment. It alleviates manual labor intensity to some extent while improving operational stability and reliability. The proposed method offers practical significance for advancing intelligent transformation and upgrading in industrial applications, such as logistics.

## 2. Materials and Methods

### 2.1. Active Binocular Vision System

#### Laser-Assisted Measurement Principle

**Figure 3:**
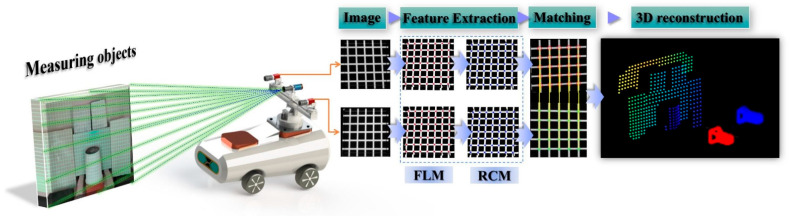
Overall Workflow of the Measurement System.

**Figure 4:**
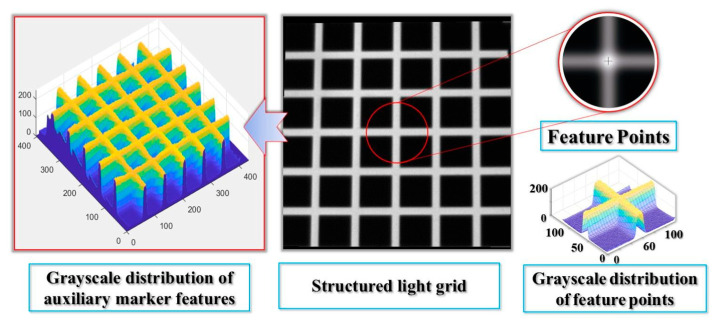
Construction of the Feature Point Pattern.

To address the challenges of feature extraction and 3D reconstruction in weak-texture scenes, where texture features are limited or unevenly distributed, a solution integrating laser marking and binocular vision is proposed. As shown in Figure 3, the system consists of a small laser projector and a binocular camera. The laser projector projects a marking pattern onto the environment (Figure 4), while the binocular camera captures the feature points. By leveraging the collimation and directionality of the laser, the system accurately extracts the sub-pixel center at the intersection of the horizontal and vertical light strips. To balance both efficiency and accuracy, the feature point extraction process is divided into two steps: first, morphological features and edge gradient constraints are used to quickly locate the feature points and determine their initial positions; second, sub-pixel precision is achieved by considering the distribution characteristics of the light strips. Finally, a sparse point cloud is generated for environmental modeling through epipolar constraint matching and parallax re-construction.

In prior work, the equipment system was precisely calibrated using Zhang’s calibration method. Rigid connections between devices ensure stability and reliability, with periodic recalibration performed to maintain accuracy. For handling high reflectivity, suppression is achieved through optical filtering (by adding a filter in front of the camera lens) combined with digital filtering (BSCB-Based Adaptive Tangential Compensation).

This solution, characterized by a compact structure and low equipment requirements, enhances feature information via laser-assisted marking and restores occluded parts through horizontal and vertical connections, effectively handling occlusion and lighting interference. It delivers high recognition accuracy, robustness, and cost-effectiveness. The resulting 3D scene data supports robotic tasks such as obstacle avoidance, grasping, and transportation, significantly improving operational accuracy and system reliability while reducing labor demands and errors. Its adoption will advance workflow automation and intelligence, enabling sustainable operation.

### 2.2. An Improved SURF Algorithm for Feature Point Fast Localization Method (FLM) Based on Multi-Constraints

To quickly locate the initial positions of the auxiliary marker feature points, an improved SURF algorithm for feature point FLM based on multi-constraints has been proposed. The algorithm incorporates grayscale and edge gradient constraints into the SURF algorithm to enhance its discriminative capability and constructs a multivariate constraint discriminant to quickly identify the initial positions. Additionally, a redundancy removal model based on feature point distribution information is introduced to eliminate duplicate data, achieving effective data refinement and reducing the overall data volume.

#### 2.2.1. Multivariate Constrained Discriminant Construction

To effectively locate the initial position of feature points and reduce invalid data, the SURF extraction discriminant method was used. A multi-constraint discriminant was established.as shown in Equation (1):

(1) $$\det(H)=\frac{\left(\sqrt{G_{x^{\prime }}^{2}+G_{y^{\prime }}^{2}}-T\right)}{\left|(\sqrt{G_{x^{\prime }}^{2}+G_{y^{\prime }}^{2}}-T)\right|}\times (D_{xx}+D_{yy})\times (D_{xx}D_{yy}-{\left(0.9D_{xy}\right)}^{2})$$

In the equation, The determinant det(*H*) reflects the rate of change in the local region of the image at the point $P(x^{\prime },y^{\prime })$, $D_{xx}=\frac{\partial^{2}I}{\partial x^{2}}$ and $D_{\mathrm{yy}}=\frac{\partial^{2}I}{\partial y^{2}}$ represents the second-order partial derivatives of the filter in the *x* and *y* directions of the image $P(x^{\prime },y^{\prime })$, respectively, *T* is the threshold (set to 1 after binarization), $G_{x}$ and $G_{y}$, represents the horizontal and vertical convolution kernels, are:

(2) $$G_{x\prime }=\left[\begin{array}{ccc} -1 & 0 & +1\\ -2 & 0 & +2\\ -1 & 0 & +1 \end{array}\right]*I(x^{\prime },y^{\prime })$$

(3) $$G_{y\prime }=\left[\begin{array}{ccc} +1 & +2 & +1\\ 0 & 0 & 0\\ -1 & -2 & -1 \end{array}\right]*I(x^{\prime },y^{\prime })$$

This discriminant can accurately extract feature points while significantly reducing invalid data generation, thereby improving the system’s feature extraction efficiency.

#### 2.2.2. Multivariate Constraint Model Based on Feature Point Position and Grayscale Characteristics

**Figure 5:**
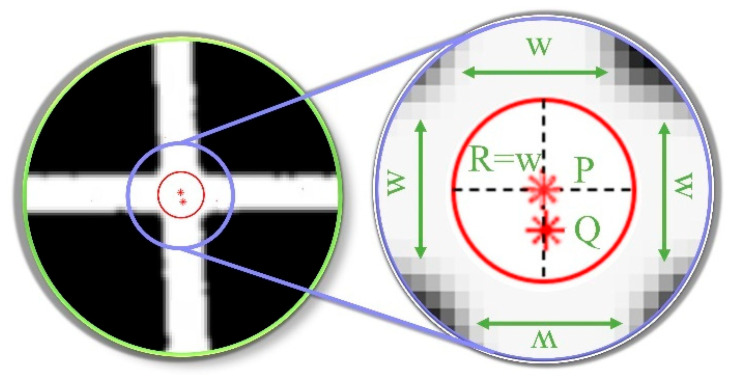
Workflow for Screening Unique Feature Points.

Given the redundancy caused by repeated feature points at the feature points shown in Figure 5 (W refers to the fringe width, R denotes the search radius, and the red asterisk-marked points P and Q represent the positions of feature points extracted by FLM), the △f(x,y) discriminant based on the acquisition of feature point distribution information is introduced, and a redundant removal model is proposed to obtain the final feature point P(x,y), as shown below.

(4) $$P(\bar{x},\bar{y})=P(\bar{x}=\frac{\sum_{i=1}^{n}△f(x,y)\cdot x_{i}}{\left|Q_{valid}\right|},\bar{y}=\frac{\sum_{i=1}^{n}△f(x,y)\cdot y_{i}}{\left|Q_{valid}\right|})$$

In the equation, *n* is the total number of candidate points, $\left|Q_{valid}\right|$ is the number of redundant points whose distance is less than the light strip width *w* near point *P*, and △f(x,y) is the discriminant, which takes the value of 1 when it is less than the light strip width *w*, otherwise it takes the value of 0:

(5) $$△f(x,y)=\left\{\begin{array}{cc} 0 & \sqrt{\sum_{i=1}^{n}\left(P_{\left(x,y\right)}-Q_{\left(x_{i},y_{i}\right)}\right)}>w\\ 1 & \sqrt{\sum_{i=1}^{n}\left(P_{\left(x,y\right)}-Q_{\left(x_{i},y_{i}\right)}\right)}<w \end{array}\right.$$

The redundant point removal model can effectively eliminate duplicate valid data, reducing data volume and shortening the time required for subsequent processing.

#### 2.2.3. Rapid Positioning and Verification of Feature Point Initial Position

**Figure 6:**
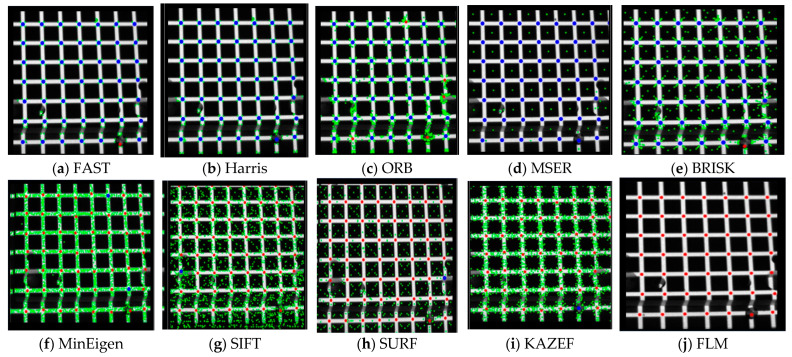
Comparison of Feature Point Extraction with Various Corner Detectors.

To verify the effectiveness of the proposed FLM, it is compared with traditional corner point extraction algorithms, such as SURF and Harris, on the same image set. As shown in Figure 6, invalid points are marked in green, missed points in blue, and successfully extracted points in red.

As shown in Figure 6, the traditional method generates many invalid points, indicated by green points. Similarly, algorithms such as Harris, MSER, and FAST also produce many missed points, indicated by blue points. In contrast, the proposed method accurately extracts feature point locations, as indicated by red points, and effectively eliminates invalid data.

Multiple methods were used to extract features from the same image (Containing 208 intersection points of the horizontal and vertical light strips, which are the actual feature points), and the results are shown in Table 2. The “Total” column represents the total number of feature points extracted by each method, including both valid and invalid points. The “Effective extraction number” column shows the number of valid data points, and the effective extraction rate (%) is calculated based on the proportion of valid points to the num of actual feature points. As shown in the table, although methods such as SIFT, SURF, and KAZE have high extraction rates, they are slower and generate more invalid data. Methods like ORB and FAST are faster but have lower effective extraction numbers, failing to meet the requirements. In contrast, the FLM demonstrates higher accuracy in extracting feature points at grid intersections, generates fewer invalid data, and achieves a better balance between extraction speed and accuracy.

**Figure 7:**
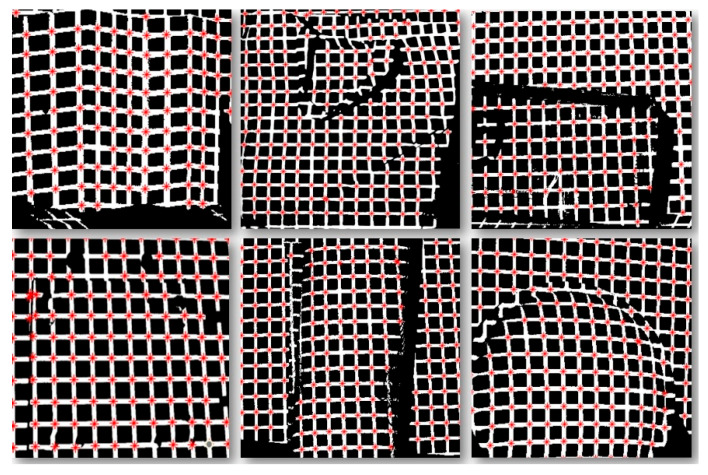
Fast Localization Results of Feature Points on Various Surfaces.

To evaluate the extraction performance of the FLM on surfaces of various shapes, rapid localization experiments of auxiliary marker feature points were conducted in environments containing a variety of objects, including curved, folded, and flat surfaces. The extraction results of the FLM are indicated by the red dots in the Figure 7 below:

The results after the initial position extraction of the feature points are statistically analyzed, as shown in the following Table 3:

As shown in Figure 7, the red dots indicate that despite the challenges posed by low-texture objects, diverse surface shapes, and interference factors such as occlusion, stacking, and deformation, the proposed method can still accurately extract the positions of surface feature points. Combined with the statistical results in Table 3, the “Total” column represents the total number of feature points extracted by the FLM, the “Valid points” column represents the number of valid points extracted by the FLM, and the “Actual feature points” column represents the total number of valid feature points in the current experiment (Number of intersection points of horizontal and vertical light strips). The ratio of “Valid points” to “Actual feature points” is the Extraction rate (%). It can be seen that even under different surface and interference conditions, the proposed method’s extraction accuracy remains above 96%, thus meeting the robot’s requirements for both extraction speed and accuracy.

### 2.3. An Robust Correction Method (RCM) for Feature Points Based on Light Strip Grayscale Consistency

To improve the accuracy and robustness of feature point extraction, an RCM is proposed that combines the Steger algorithm with a weighted fitting model. This method is based on the initial positions of the feature points obtained in the previous step, as well as the principles of laser imaging and optical characteristics. The method first uses the Steger algorithm to extract the center point set of the horizontal and vertical light strip intersections at the feature point and then calculates the precise position of the feature point through the weighted fitting model, thereby enhancing the accuracy of feature point extraction. Even when the local light strip is distorted or occluded, this method can still extract feature points using information from other horizontal and vertical light strips, ensuring that the robot meets the high precision and robustness requirements for feature point extraction in tasks such as grasping.

#### 2.3.1. Analysis of Laser Strip Cross-Section Characteristics

**Figure 8:**
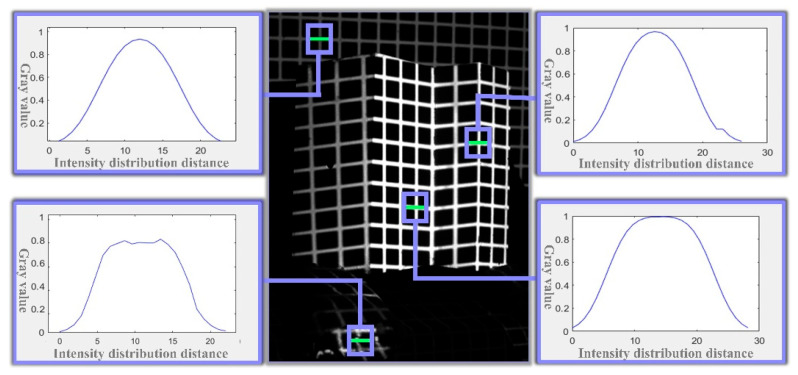
Grayscale Profile of the Laser Light Strip Cross-Section.

To ensure the accuracy and robustness of the feature point extraction process, the proposed method analyzes the grayscale distribution of the laser stripe based on the principle of laser luminescence. As illustrated in Figure 8, the grayscale distribution remains stable under various conditions, including overexposed mirror reflections, foreground highlights, and dark background regions. Specifically, the distribution follows a Gaussian profile, is uniformly extended along the length of the stripe, and peaks at the center. The center points of the stripe’s cross-section accurately represent its central axis.

For robustness, blocking any part of a cross-section, except for the peak area, will not affect the grayscale distribution. Similarly, along the length of the strip, even if part of the cross-section is blocked, the grayscale distribution characteristics of the remaining section remain unchanged.

Additionally, considering the directionality and collimation of the light strips, as well as the invariance of line intersections under perspective projection, the intersection of the center points of the horizontal and vertical light strips represents the sub-pixel accurate position of the feature point.

#### 2.3.2. Laser Stripe Cross-Section Center Extraction Algorithm

##### Cross-Section Center Extraction

The Steger algorithm extracts sub-pixel center point sets of horizontal and vertical laser stripes through Hessian eigenvalue analysis, providing input data for subsequent feature point localization by fitting horizontal and vertical stripe point sets.

**Figure 9:**
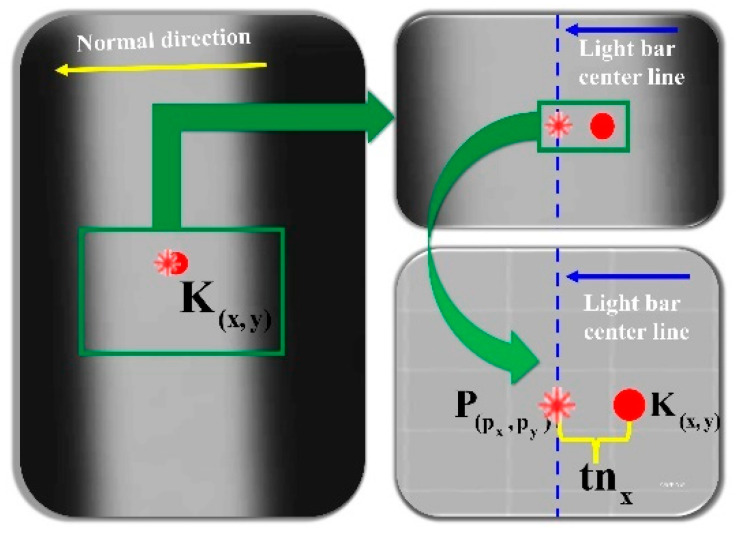
Steger-Based Centerline Extraction from Laser Stripe Cross-Section.

As shown in Figure 9, the sub-pixel center point coordinates $P_{\left(p_{x},p_{y}\right)}$ of the light strip cross-section are extracted based on the cross-section where point $K_{\left(x,y\right)}$ is located:

(6) $$P(p_{x},p_{y})=(x+tn_{x},y+tn_{y})$$

In the equation, (*x*, *y*) represents point *K*’s initial coordinates, $n=(n_{x},n_{y})$ is represented by the eigenvector corresponding to the maximum eigenvalue of the Hessian matrix, that is, the normal direction of the light strip, *t* is the sub-pixel offset calculated through:

(7) $$t=-\frac{{n^{2}}_{x}D_{x}{+n}_{y}n_{x}D_{y}}{{n^{2}}_{x}D_{xx}{+2n}_{x}n_{y}D_{xy}+{n^{2}}_{y}D_{yy}}$$

with $D_{x}$ and $D_{y}$ being first-order and $D_{xx}$, $D_{xy}$, $D_{yy}$ second-order image derivatives at *K*.

**Figure 10:**
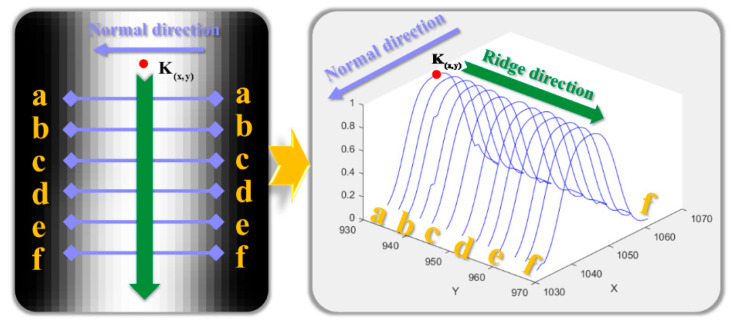
Schematic Diagram of Cross-Section Center Point Extraction Using the Steger Method.

As shown in Figure 10, starting from the center point $P_{\left(p_{x},p_{y}\right)}$ of the cross-section, the search is advanced along the ridge line direction to obtain the center points of other cross-sections of the light strip to improve efficiency.

**Figure 11:**
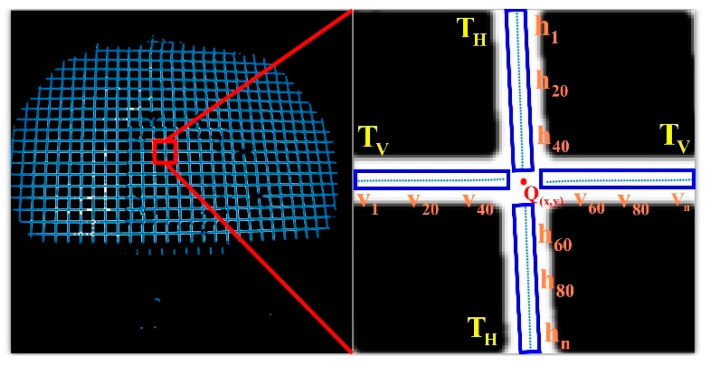
Cross-section center point extraction diagram.

After obtaining the center point $P_{\left(p_{x},p_{y}\right)}$ of the light strip cross-section at point $K_{\left(x,y\right)}$, the search is advanced along the ridge line to obtain the center points of other cross-sections of the light strip, as shown in the following Figure 10:

Steger is used to quickly extract the centers of all light strip cross-sections from the entire image, thereby obtaining an accurate set of center point coordinates for all light strip cross-sections, as shown in Figure 11 (The blue dots in the figure represent the extracted center points of the fringe cross-sections).

##### Get Point Set

**Figure 12:**
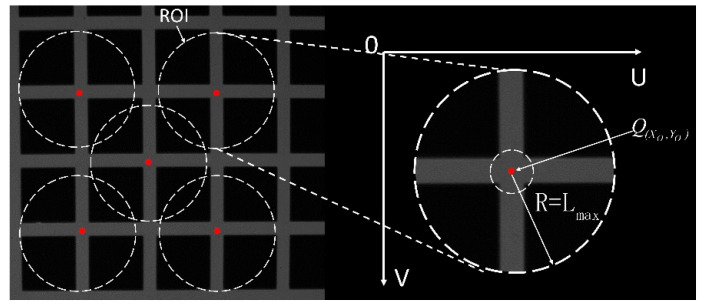
ROI region of interest.

Based on the initial position of the feature points, to deal with the loss or deformation of some sections that may be caused by local light strip occlusion and distortion, a circular area of interest with a grid width of $L_{\max}$ is defined as the radius. $L_{\max}$, as shown in Figure 12 (ROI refers to the Region of Interest in the RCM algorithm, and R denotes the radius of the ROI.) to obtain the feature points. Set the center points of the horizontal and vertical light strips in the area of interest to reduce randomness and enhance the accuracy and robustness of the fitting. As shown in Figure 11, taking the initial feature point.

As an example, Steger extracts the center of the horizontal and vertical light strip section at $Q_{{(X}_{O}{,Y}_{O})}$ to form a longitudinal point set $T_{H}$ and a transverse point set $T_{V}$.

##### Establishing Horizontal and Vertical Light Strip Fitting Model

To obtain the precise position of the feature point, a horizontal and vertical light strip fitting model is constructed based on the center points of the light strip cross-sections at feature point $Q_{{(X}_{O}{,Y}_{O})}$ obtained in the previous step, as follows:

(8) $$\left\{\begin{array}{l} {f(X}_{T_{H}})={a^{H}}_{1}+{a^{H}}_{2}X_{T_{H}}+{a^{H}}_{3}{X_{T_{H}}}^{2}+{a^{H}}_{4}{X_{T_{H}}}^{3}+{a^{H}}_{5}{X_{T_{H}}}^{4}+{a^{H}}_{6}{X_{T_{H}}}^{5}\\ {f(Y}_{T_{V}})={a^{V}}_{1}+{a^{V}}_{2}Y_{T_{V}}+{a^{V}}_{3}{Y_{T_{V}}}^{2}+{a^{V}}_{4}{Y_{T_{V}}}^{3}+{a^{V}}_{5}{Y_{T_{V}}}^{4}+{a^{V}}_{6}{Y_{T_{V}}}^{5} \end{array}\right.$$

In the equation, $X_{T_{H}}{=[X}_{1}\ldots X_{n}]$ and $Y_{T_{V}}{=[Y}_{1}\ldots Y_{V}]$ are the center point sets of the horizontal and vertical light strip cross-sections; ${a^{V}}_{\mathrm{xs}}{{=[a}^{V}}_{1}\ldots {a^{V}}_{6}{]}_{\mathrm{xs}=1,2\ldots 6}$ and ${a^{H}}_{\mathrm{xs}}{{=[a}^{H}}_{1}\ldots {a^{H}}_{6}{]}_{\mathrm{xs}=1,2\ldots 6}$ are the horizontal and vertical fitting model coefficients.

##### Constructing a Loss Function Evaluation Model

To determine the optimal model coefficients, a loss function is established as follows:

(9) $$\left\{\begin{array}{l} {{L(a}^{H}}_{\mathrm{xs}})=\sum_{T_{H}=1}^{n}W_{\mathrm{qz}}{{(Y}_{T_{H}}{\mathrm{-f}(X}_{T_{H}}))}^{2}\\ {{L(a}^{V}}_{\mathrm{xs}})=\sum_{T_{V}=1}^{n}W_{\mathrm{qz}}{{(Y}_{T_{V}}{\mathrm{-f}(X}_{T_{V}}))}^{2} \end{array}\right.$$

In the equation, $W_{\mathrm{qz}}{=[W}_{1}\ldots W_{n}]$ is the weight, By assigning greater weight to the center points closer to the feature point and less weight to those farther away during the process, $X_{V}{=f(Y}_{T_{V}})$ and $y_{H}{=f(X}_{T_{H}})$ are the horizontal and vertical observation values of each point obtained by fitting the model.

By combining the fitting model constructed with the center point sets of the horizontal $X_{T_{v}}{=[X}_{1}\ldots X_{n}]$ and vertical $Y_{T_{H}}{=[Y}_{1}\ldots Y_{n}]$ light strips, the coordinates of the intersection of the center lines $Q_{{(X}_{a}{,Y}_{a})}$ of the horizontal and vertical light strips can be obtained, as shown in Figure 12, and it is extracted as the precise position of the feature point.

#### 2.3.3. Experiment on Accurate Feature Point Acquisition

To verify the RCM, experiments are conducted to accurately locate auxiliary marker feature points on various typical environmental objects, including curved surfaces, folded surfaces, and flat planes.

**Figure 13:**
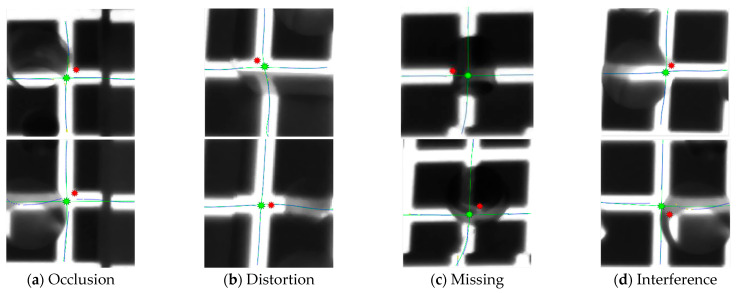
Comparison chart of Fast localization and Robust correction.

Figure 13 shows the extraction results of FLM (red points) and RCM (green points) under the influence of interference factors such as light strip occlusion, surface protrusions, missing segments, distortions, or discontinuities. The green line represents the fitted curve, blue points indicate the center points of the light strip cross-sections, and yellow points highlight outliers automatically removed by the algorithm. As seen in the figure, the feature points extracted by RCM (green points) are closer to the standard position (the center of the intersection of the horizontal and vertical light strips) compared to the initial positions determined by FLM (red points). This demonstrates that RCM has certain anti-interference capabilities and provides higher accuracy than FLM.

Table 4 presents 10 randomly selected sets of data from multiple FLM and RCM comparison experiments. The data show that the average error and variance of RCM are significantly smaller than those of FLM, which suggests that RCM achieves higher precision.

Based on the comparison results from Figure 13 and Table 4, it is evident that even when feature points are partially missing, the RCM algorithm can still utilize information from surrounding areas to perform reconstruction, demonstrating strong anti-interference capabilities and robustness.

#### 2.3.4. Matching and Reconstruction

**Figure 14:**
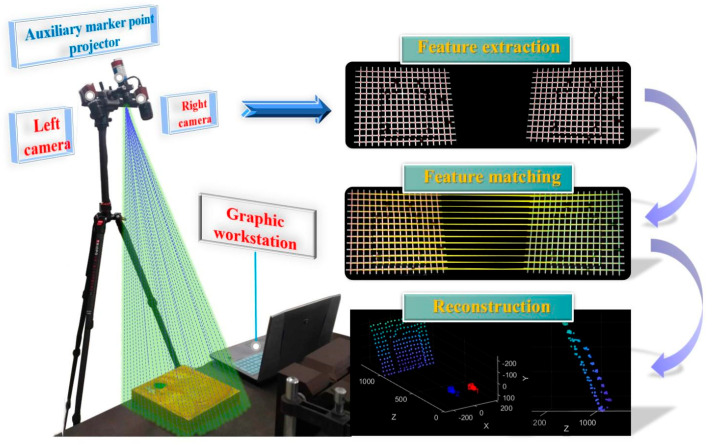
Point Cloud Reconstruction and Image Fitting Using Auxiliary Markers.

Given the well-defined correspondence between feature points in the images captured by the binocular camera, and the advantages of the epipolar constraint matching algorithm in terms of simplicity, speed, and high precision, this algorithm is used to match feature points between the left and right images. Combined with the stereoscopic vision parallax method, it is then applied to 3D reconstruction. The reconstruction results are shown in the Figure 14 below.

## 3. Results

To verify the effectiveness of the proposed method, accuracy verification and complex environment restoration experiments were conducted using standard plates and in complex environments.

The experiment was conducted on a system with an Intel (Santa Clara/CA/USA) i7-8750H CPU (2.2 GHz), 8 GB RAM, and Windows 10, using MATLAB R2013a. A 500 nm grid laser projector (OPTO LTRPHP3W, Mantova/LO/Italy) was used for projection, and images were captured by an Allied (Stadtroda/TH/GermanyGermany) G-419B monochrome camera (2048 × 2048 px) equipped with a 520 nm filter to reduce glare.

### 3.1. Accuracy Verification Experiment

**Figure 15:**
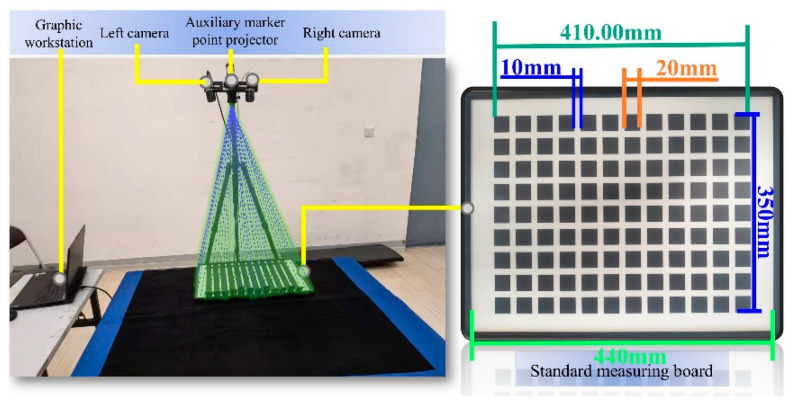
Validation Test Bench for Auxiliary Marker Restoration.

**Figure 16:**
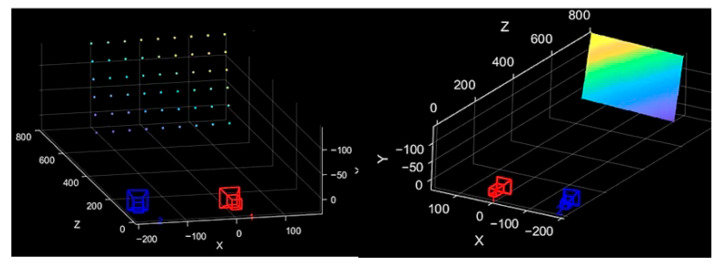
Point cloud and fitting image after auxiliary marker reconstruction.

This experiment uses a high-precision standard plate with an error margin of 0.01 mm to verify the accuracy of auxiliary feature point extraction and 3D reconstruction. The experimental setup is shown in the Figure 15 below:

The sparse point cloud and reconstructed image of the standard plate are generated using the feature point extraction and reconstruction method proposed, as shown in Figure 16 (In the figure, the camera baseline is used as the x-axis. The closer the reconstructed coordinates are to the x-axis, the yellower they appear; conversely, the farther they are, the bluer they appear).

**Figure 17:**
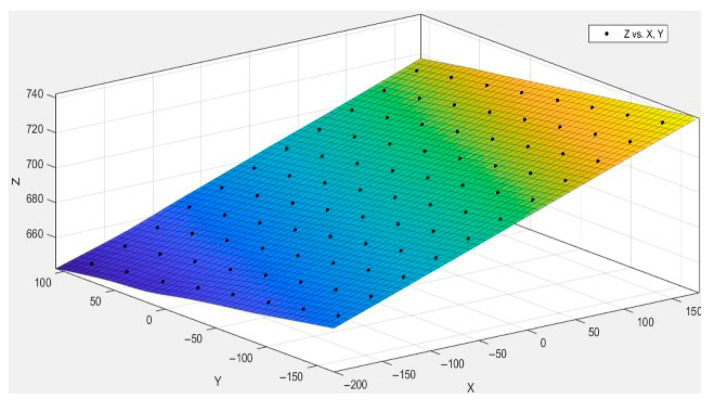
Feature point 3D plane fitting diagram.

**Figure 18:**
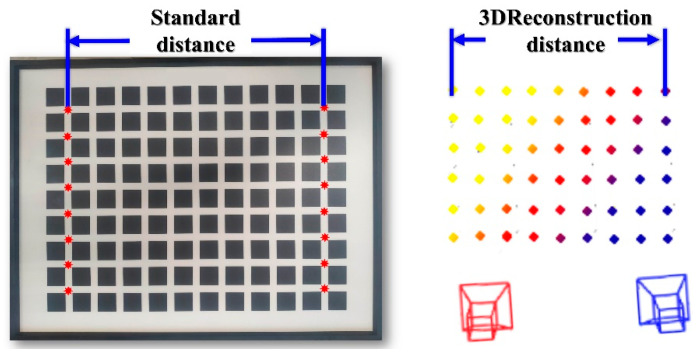
Schematic Diagram of Feature Point Reconstruction Accuracy Verification.

The 3D plane fitting was performed based on the reconstructed 3D coordinates of the feature points, with the fitting result shown in Figure 17. The black points in the figure represent the 3D coordinates of the feature points, all of which are essentially located on the same plane. The residual sum of squares (SSE) of the fitted 3D plane is $1.925\times 10^{-24}$, and the coefficient of determination is 1, indicating that the 3D reconstruction of the feature points has a high degree of planarity. This result is consistent with the actual condition of the standard measurement plate, further validating the superiority of the overall reconstruction performance. To further evaluate the reconstruction accuracy, we compared the actual distances of the first and last columns of the standard plate with the 3D reconstruction results. The error information is shown in Figure 18.

It is demonstrated through experiments that the auxiliary marker point restoration method can accurately determine the 3D coordinates of the auxiliary marker points. The system’s absolute error is less than 0.5 mm, and the relative error is below 2‰(as shown in Table 5), meeting the accuracy requirements for mobile robot environment modeling and measurement tasks.

### 3.2. Three-Dimensional Reconstruction Experiment in Complex Environment

**Figure 19:**
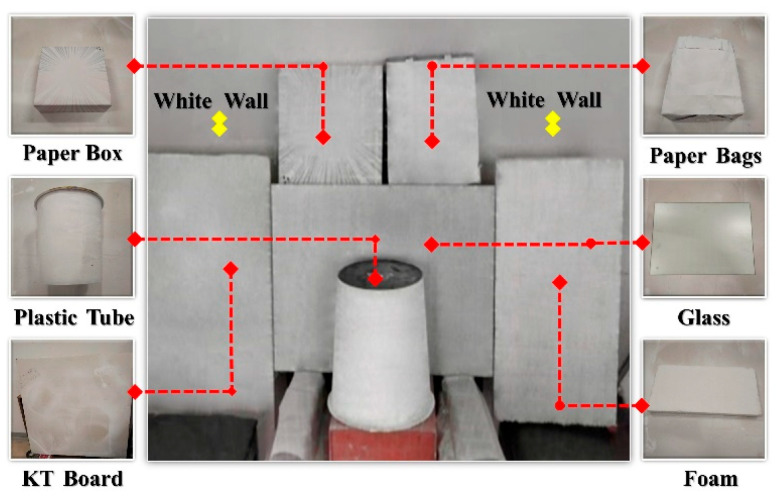
Complex environment reconstruction experimental scene diagram.

Various objects, including paper boxes, paper bags, plastic drums, glass, foam, and KT boards, as well as other items with diverse shapes and textures, are utilized to create a complex test environment in the industrial logistics scenario, as shown in Figure 19 (Yellow marks indicate the white wall positions). Under normal, dim, and no-light conditions, the measurement system developed in this study is used to collect images, and the proposed method is employed for feature extraction and reconstruction.

**Figure 20:**
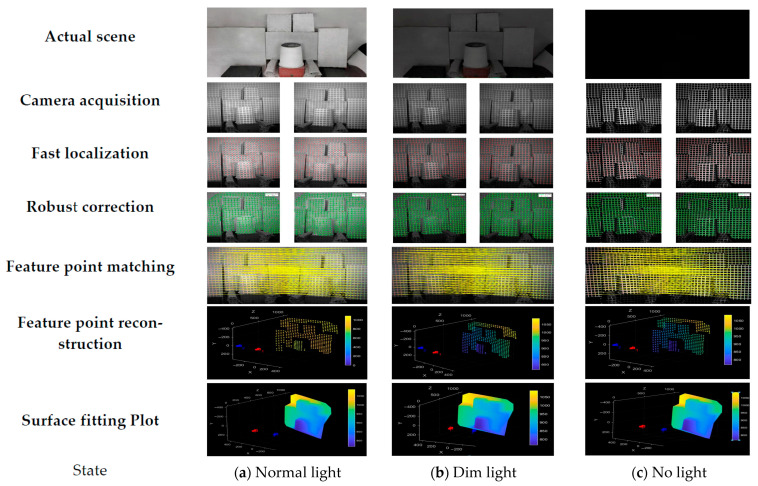
Feature extraction and image reconstruction in complex environments.

As shown in Table 6 and Figure 20, the weak-texture environment modeling method effectively handles different materials, lighting conditions, and surface morphologies, demonstrating strong anti-interference capability. With a feature extraction rate exceeding 97%, this method meets the requirements for environment modeling in weak-texture scenarios.

### 3.3. Three-Dimensional Reconstruction During Motion

**Figure 21:**
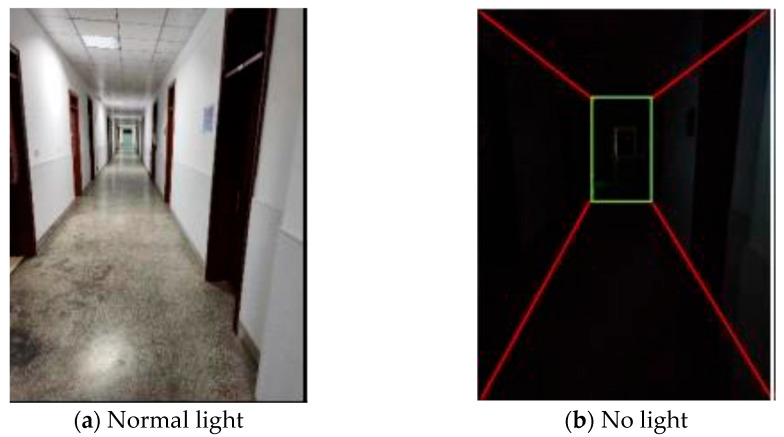
Experimental Scenario Diagram.

**Figure 22:**
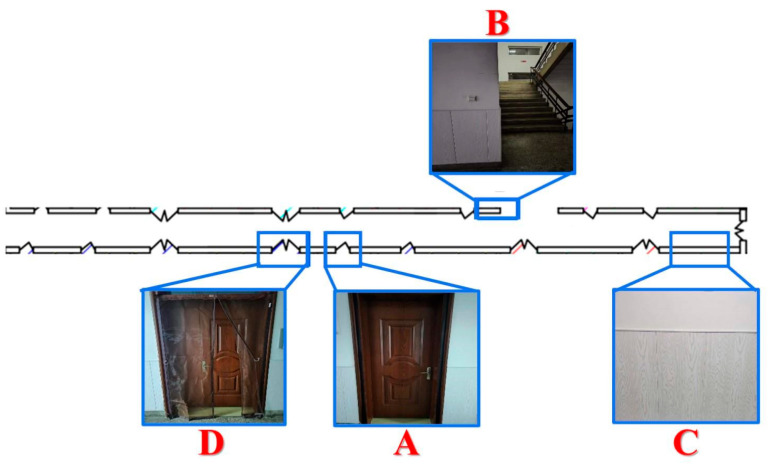
Structural Diagram of the Experimental Scenario.

To validate the effectiveness of the proposed approach for mobile environment modeling in weak-texture scenarios, a typical narrow corridor with a depth of 50 m and a width of 2 m was selected as the experimental site. The experimental environment is illustrated in Figure 21, comprising four local areas labeled A, B, C, and D. As shown in Figure 22, area A corresponds to a narrow doorway, area B to a staircase corner, area C to a corridor plane, and area D to a wide doorway.

**Figure 23:**
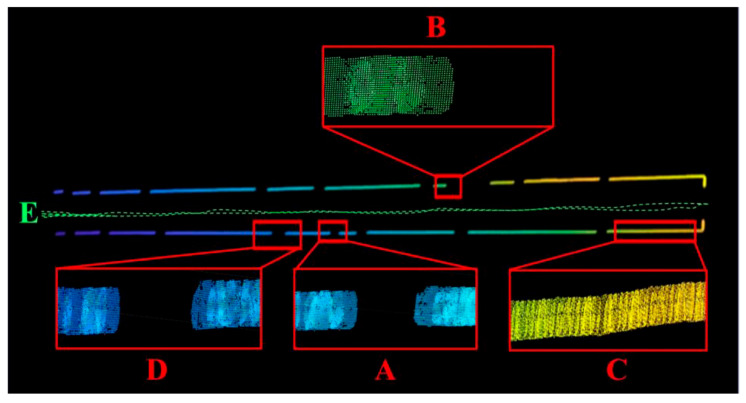
Three-Dimensional Reconstruction of the Experimental Scene under Motion.

The experimental results are shown in Figure 23. Using the proposed method, the environment was modeled in a mobile state and visualized. The green dashed line E in the figure represents the actual trajectory of the robot. Enlarged views of the four local areas A, B, C, and D clearly depict the passage spaces of a narrow doorway, a staircase corner, a corridor plane, and a wide doorway, respectively, with the overall geometry closely matching that of the real scene. These results demonstrate that the proposed environment modeling method maintains excellent accuracy and robustness even under weak-texture and dynamic scene conditions.

### 3.4. Limitations and Future Work

#### 3.4.1. Limitations

The method is suitable for normal lighting conditions (e.g., indoor incandescent lights and daylight) and low-light or dark environments. It is designed for robots with low to moderate speeds, enabling effective perception and modeling in weak-texture settings. However, its performance may be limited by strong light interference, translucent objects, or surfaces with very low reflectivity, such as super-black materials.

#### 3.4.2. Future Work

1. To enhance environment modeling under interference, future work will explore deep learning-based image quality enhancement and marker feature restoration.
2. While the current method balances efficiency and accuracy by extracting only point features, future research will incorporate line features to further improve environmental modeling and perception.
3. There is significant potential to improve system efficiency. Future efforts will focus on algorithm optimization, GPU parallelization, and C++ code restructuring to achieve a 5–8× speedup in 3D reconstruction, enabling real-time 3D perception for robotic applications.

## 4. Conclusions

The proposed solution addresses key challenges in weak-texture environments, such as limited feature extraction, balancing efficiency and accuracy, large data volumes, and low interference resistance. By combining laser-assisted marking and binocular vision, it enhances environmental perception. Laser marking boosts feature visibility, while the FLM and RCM algorithms enable rapid, accurate feature extraction. The reconstruction process generates evenly distributed 3D data to support robotic perception.

Compared to traditional methods, this solution offers several advantages: low cost (only $1400 USD), low computational complexity, compact design, fast processing, high accuracy, and strong interference resistance. It effectively prevents common issues in weak-texture environments, such as collisions, product damage, misplacement, and pose estimation errors, which result from incomplete 3D data. This reduces misclassification and rework, translating into cost savings, minimized downtime, and improved operational efficiency. The low equipment cost also accelerates ROI, making the system affordable for small businesses with limited budgets.

This method provides an economical, compact, and adaptable solution for automation in weak-texture environments. Experimental results confirm its effectiveness, highlighting its potential in industrial applications, particularly in logistics, warehousing, and manufacturing, where weak-texture environments are prevalent. It can significantly boost efficiency and reduce operational costs.

## Figures and Tables

**Table 1:** Three-dimensional Equipment Specification Comparison Table.

Company/Country/City	Model	Device	Price (USD)	Working Distance	Accuracy
GOM GmbH/Germany/Braunschweig	GOM Scan 1	1 × LiDAR, 5 × RGB Camera	$27,000	0.3–1.0 m	0.037 mm
JOYCITY Corporation/South Korea/Seongnam	Freestyle 2	1 × LiDAR, 3 × RGB Camera,	$12,000	0.1–0.5 m	0.50 mm
Leica Geosystems AG/Switzerland/Heerbrugg	Leica BLK360 G2	1 × SL Projector, 2 × RGB Camera	$19,500	0.3–60 m	4.00 mm
Shining 3D Technology Co., Ltd. (China)/China/Hangzhou	KSCAN-Magic II	1 × SL Projector, 2 × RGB Camera, 1 × IR Camera	$24,800	0.25–1.2 m	0.020 mm
-	Present Study	1 × SL Projector, 2 × mono CMOS	$1400	0.2–2 m	0.5 mm

**Table 2:** Auxiliary Marker Feature Point Extraction Results Using Various Corner Detection Algorithms.

Method	Total	Effective Extraction Number	Effective Extraction Rate (%)	Takes Time (s)
Harris	1105	0	0	0.37
MSER	4062	3	1.4	1.13
FAST	515	2	0.9	0.04
BRISK	3551	10	1.8	0.20
ORB	10,326	19	9.1	0.08
SIFT	13,606	203	97.6	0.19
KAZEF	9662	200	96.1	1.2
SURF	2035	203	97.6	0.18
MinEigen	7231	178	85.57	0.90
FLM	206	206	99.0	0.13

**Table 3:** Initial Position Statistics Based on Fast Feature Point Extraction.

Frequency	Total	Valid Points	Actual Feature Points	Extraction Rate (%)
1	413	412	429	96.03
2	658	658	669	98.35
3	480	480	492	97.5
4	490	490	507	96.54
5	419	419	428	97.85
6	449	449	460	97.55

**Table 4:** Error Comparison Between FLM and RCM.

Num	RCM			FLM		
	Horizontal Error	Vertical Error	Euclidean Distance	Horizontal Error	Vertical Error	Euclidean Distance
1	1.41 px	0.42 px	1.47 px	7.45 px	5.33 px	9.16 px
2	1.77 px	1.63 px	2.40 px	3.69 px	0.25 px	3.69 px
3	1.15 px	0.33 px	1.19 px	4.98 px	3.15 px	5.89 px
4	1.54 px	0.21 px	1.55 px	1.10 px	5.12 px	5.09 px
5	0.60 px	0.1 px	0.60 px	2.14 px	3.14 px	3.60 px
6	2.35 px	0.03 px	2.35 px	1.28 px	5.03 px	5.09 px
7	2.09 px	0.31 px	2.11 px	2.12 px	7.20 px	7.28 px
8	0.62 px	2.02 px	2.11 px	4.04 px	2.10 px	4.47 px
9	1.46 px	0.17 px	1.46 px	3.21 px	3.03 px	4.24 px
10	1.39 px	0.16 px	1.39 px	3.13 px	5.06 px	5.83 px
Average error	1.43 px	0.5382 px	1.66 px	3.21 px	3.87 px	5.43 px
Square difference	0.31 px	0.48 px	0.32 px	3.86 px	3.82 px	2.96 px

**Table 5:** Table of Reconstruction Errors in Accuracy Validation.

Number of Trials	Actual Distance (mm)	Measurement Distance (mm)	Absolute Error (mm)	Relative Error (‰)	FLM Time (s)	RCM Time (s)	3D Reconstruction Time (s)
1	350	349.73	0.2626	0.75	0.13	0.37	0.04
2	350	349.63	0.3693	1.05	0.14	0.38	0.05
3	350	349.54	0.4545	1.29	0.12	0.39	0.08
4	350	349.76	0.2383	0.68	0.13	0.38	0.03
5	350	349.67	0.3226	0.92	0.14	0.39	0.02
6	350	349.64	0.3502	1.00	0.12	0.37	0.01

**Table 6:** Statistical Table of Extraction and Reconstruction Results in Complex Environments.

Lighting Conditions	Image	FLM		RCM		Feature Point Reconstruction	
		Feature Points	Takes Time	Feature Points	Takes Time	Feature Points	Takes Time
Normal light	Left Camera	454	0.15 s	454	0.57 s	421	0.02 s
	Right Camera	440	0.15 s	440	0.58 s		
Dim light	Left Camera	463	0.17 s	463	0.57 s	426	0.04 s
	Right Camera	468	0.17 s	468	0.59 s		
No light	Left Camera	460	0.18 s	460	0.61 s	432	0.07 s
	Right Camera	466	0.17 s	466	0.65 s		

## Data Availability

The original contributions presented in this study are included in the article. Further inquiries can be directed to the corresponding author.
